# pH and redox sensitive albumin hydrogel: A self-derived biomaterial

**DOI:** 10.1038/srep15977

**Published:** 2015-11-03

**Authors:** S Thirupathi Kumara Raja, T Thiruselvi, Asit Baran Mandal, A Gnanamani

**Affiliations:** 1 CSIR-CLRI Adyar, Chennai, Tamil Nadu, India

## Abstract

Serum albumin can be transformed to a stimuli (pH and redox) responsive hydrogel using the reduction process followed by oxidative refolding. The preparation of albumin hydrogel involves a range of concentrations (75, 150, 300, 450, 600 and 750 μM) and pH (2.0–10.0) values and the gelation begins at a concentration of 150 μM and 4.5–8.0 pH value. The hydrogel shows maximum swelling at alkali pH (pH > 9.0). The increase in albumin concentration increases hydrogel stability, rheological property, compressive strength, proteolytic resistance and rate of *in vivo* biodegradation. Based on the observed physical and biological properties of albumin hydrogel, 450 μM was determined to be an optimum concentration for further experiments. In addition, the hemo- and cytocompatibility analyses revealed the biocompatibility nature of albumin hydrogel. The experiments on *in vitro* drug (Tetracycline) delivery were carried out under non reducing and reducing conditions that resulted in the sustained and fast release of the drug, respectively. The methodology used in the preparation of albumin hydrogel may lead to the development of autogenic tissue constructs. In addition, the methodology can have various applications in tissue engineering and drug delivery.

The recent developments in biomaterial research have resulted in various innovative materials with clinical applications. However, intensive research is required for developing materials for therapeutic applications^1^. In the field of biomaterials research, hydrogels are known to deliver proteins, drugs, antibodies, DNA, growth factors and immunological molecules^2^,^3^. The preparation of hydrogels for biomedical applications depends on the functional properties, such as swelling, biocompatibility, biodegradability, non-toxicity and mechanical stability, of the materials. The various polymeric materials and cross-linking systems in hydrogels help in enhancing its delivery efficiency. Recently, stimuli-responsive hydrogels were reported to be an efficient delivery system of therapeutic molecules to the targeted site. This stimuli-responsive property can be integrated into hydrogels using appropriate functionalisation chemistry on the polymeric material^4^,^5^,^6^,^7^,^8^.

Although the pH responsive property of hydrogels has been widely exploited, its redox response property has not been much reported^9^,^10^,^11^. This redox property can be achieved through functionalisation with thiol moieties to the polymer backbone of either natural or synthetic origin^12^,^13^,^14^,^15^,^16^,^17^. Natural materials are biocompatible and biodegradable compared to synthetic polymeric materials. Proteins, a natural material, can be effectively used due to their availability and unique structural properties. Various proteins, such as collagen, gelatin and albumin, are used in tissue engineering and drug delivery. Currently, autogenic sources are being explored for protein harvesting. Of these proteins, albumin can be used in various applications as they can be easily harvested from human blood plasma.

Serum albumin, the most abundant globular protein in blood, consists of 580 amino acid residues (66 kDa) with 17 disulphide bridges and has 1 free sulphydryl group in a single polypeptide chain. The protein consists of 54% alpha helix and 40% beta structure^18^, and is about 50 g/L or two-third of plasma protein. It functions as a transport protein for numerous endogenous and exogenous substances.

Studies have reported that albumin can be transformed into a hydrogel or any other biomaterials. Serum albumin has been widely used as a backbone material in the preparation of hydrogels, microspheres, nanoparticles and scaffolds using suitable cross-linkers such as glutaraldehyde^19^,^20^,^21^. However, although the addition of glutaraldehyde to hydrogel helps in crosslinking and imparting stability, its toxicity is of concern^19^. This problem can be overcome if the innate di-sulphide bridges of proteins are exploited in a suitable way through redox chemistry. Thus, the use of external cross-linkers can be avoided and a redox responsive property can be achieved^22^,^23^,^24^,^25^,^26^.

Hence, there is a requirement for an autogenic material (i.e., a unique material derived from an individual’s blood protein–human serum albumins, HSA) in the preparation of stimuli-responsive hydrogels. Bovine serum albumin (BSA) could serve as a valuable alternate model to HSA due to its medical importance, low cost, high availability and about 76% similarity in molecular structure with HSA. Hence, in this study, we attempted to prepare pH and redox responsive self-derived BSA hydrogel using glutathione as a reducing agent. The hydrogel was further characterized for its physical and mechanical stability, biodegradability and *in vitro* and *in vivo* biocompatibility.

## Results and Discussion

**Figure 1:**
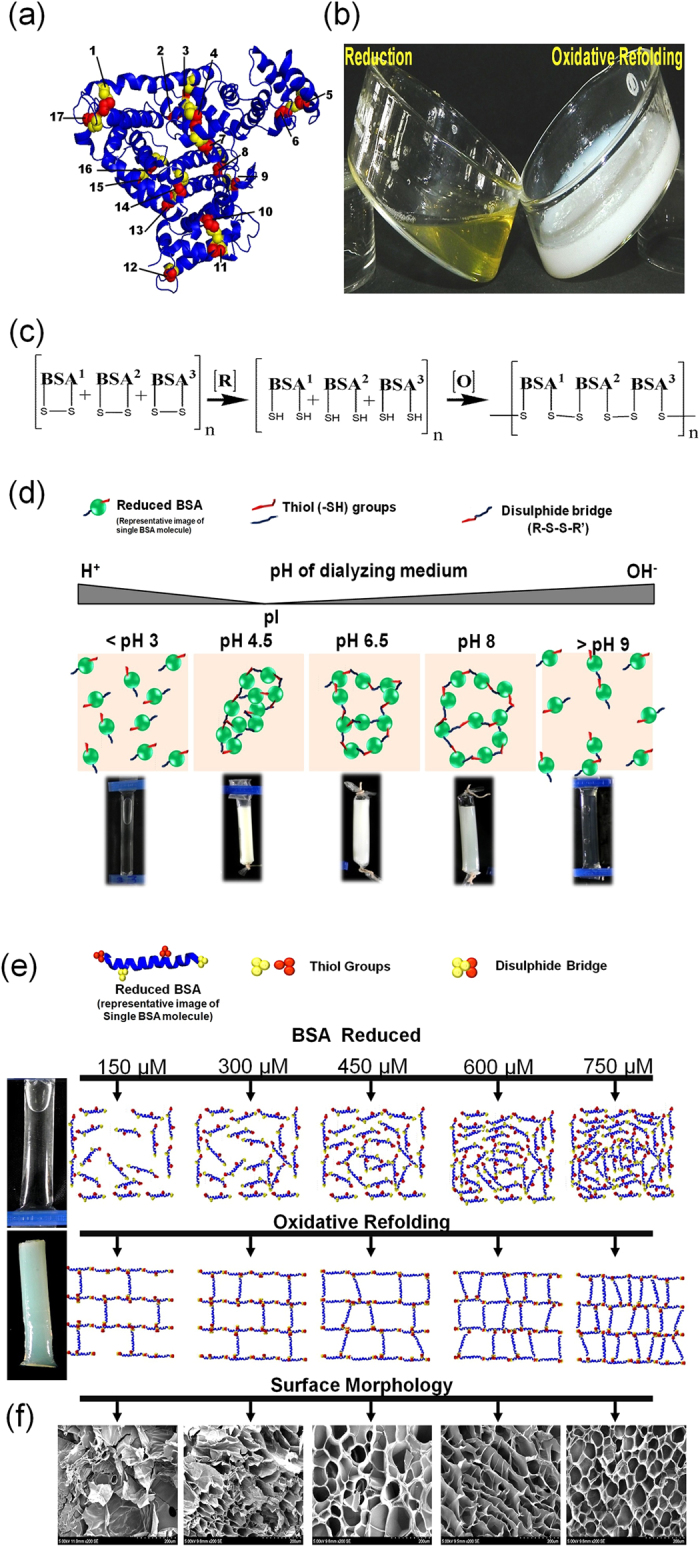
(**a**) Native BSA (3D) showing disulphide bridges (numbered) in the alpha helix region of the protein. (**b**) Physical state of glutathione reduced BSA before and after oxidation. (**c**) The equation represents the reduction of BSA and under oxidative condition it undergoes non-native disulphide bridge formation to form a polymeric network. (**d**) The scheme represents a physical observation and the molecular interaction involved in the transformation of BSA molecules at different pH. (**e**) Hypothetical representation of the reduced BSA molecules (at various concentration) undergoing oxidative refolding in non-native form and transforms into a gel. (**f**) Scanning electron micrograph of BSA gel for the selected concentrations studied.

Through a redox mechanism, the study investigated the methodology involved in the transformation of the serum albumin solution to a stimuli responsive hydrogel. BSA is a single polypeptide chain made up of 568 amino acids with 17 disulphide bridges and one free thiol group (Fig. 1a). During reduction, in the presence of reduced glutathione, the protein undergoes thiol disulphide interchange reaction. The reaction condition was maintained above the physiological pH, where the thiols in the reduced glutathione form a thiolate anion and act as a strong nucleophile, which readily undergoes a nucleophilic attack along the disulphide bridges of the protein. The cleaved disulphide bridges of the BSA undergo oxidative refolding when the reductive environment is removed from the system through dialysis (Fig. 1b). During oxidative refolding, the free thiols in the BSA undergoes disulphide bridge formation in a non- native form resulting in the formation of albumin hydrogel (Fig. 1c). There are no cross-linkers involved in this process and the hydrogel obtained is a self-derived material. The observations made in the present study have been explained in the following paragraphs.

Concentration of the serum albumin and the pH of the dialyzing medium were the rate limiting steps for the gel formation. By changing the pH of the dialyzing medium in the range of 4.0–5.0, the rate of gel formation was found to be rapid, but the gel was too rigid and brittle and this could be reasoned to the isoelectric pH of BSA (Fig. 1d). There was no gel formation at pH <3.0 and only meager intermolecular interactions occur due to the prevailing protonated conditions, which is not favorable for S-S bridge formation (Fig. 1d). At pH between 6.0–8.0, appreciable intermolecular interactions occurs and leads to quick gel formation suggested the favorable conditions for S-S formation and the availability of albumin molecules at very close proximity (Fig. 1d). With pH > 9.0, no gel formation was observed due to an increase in repulsive forces inaddition to the slow degradation of the protein (Fig. 1d)^10^.

With respect to BSA concentration, when the concentration was less than 10 μM, there was no change in the physical nature of the samples. However, when the concentration of the BSA increased from 10–100 μM, the oxidative refolding showed an aggregation of molecules. An additional increase in the BSA concentration (from 150 μM), the re-oxidation process proceeds with the gel formation. When the BSA concentration still increased further (300, 450, 600 & 750 μM), the rate of gel formation was fast (within four hours) and the mechanical stability of the gel was also found to be increased (Fig. 1e).

Scanning electron microscopic analysis depicts the surface morphology of the BSA hydrogel obtained at different concentrations (Fig. 1f). At 150 and 300 μM concentration, the hydrogel displayed improper pore structure due to insufficient protein molecules. However, when the concentration of the protein increased from 300 to 750 μM, the hydrogel shows well connected pore structure with an increase in the uniform network structure.

**Figure 2:**
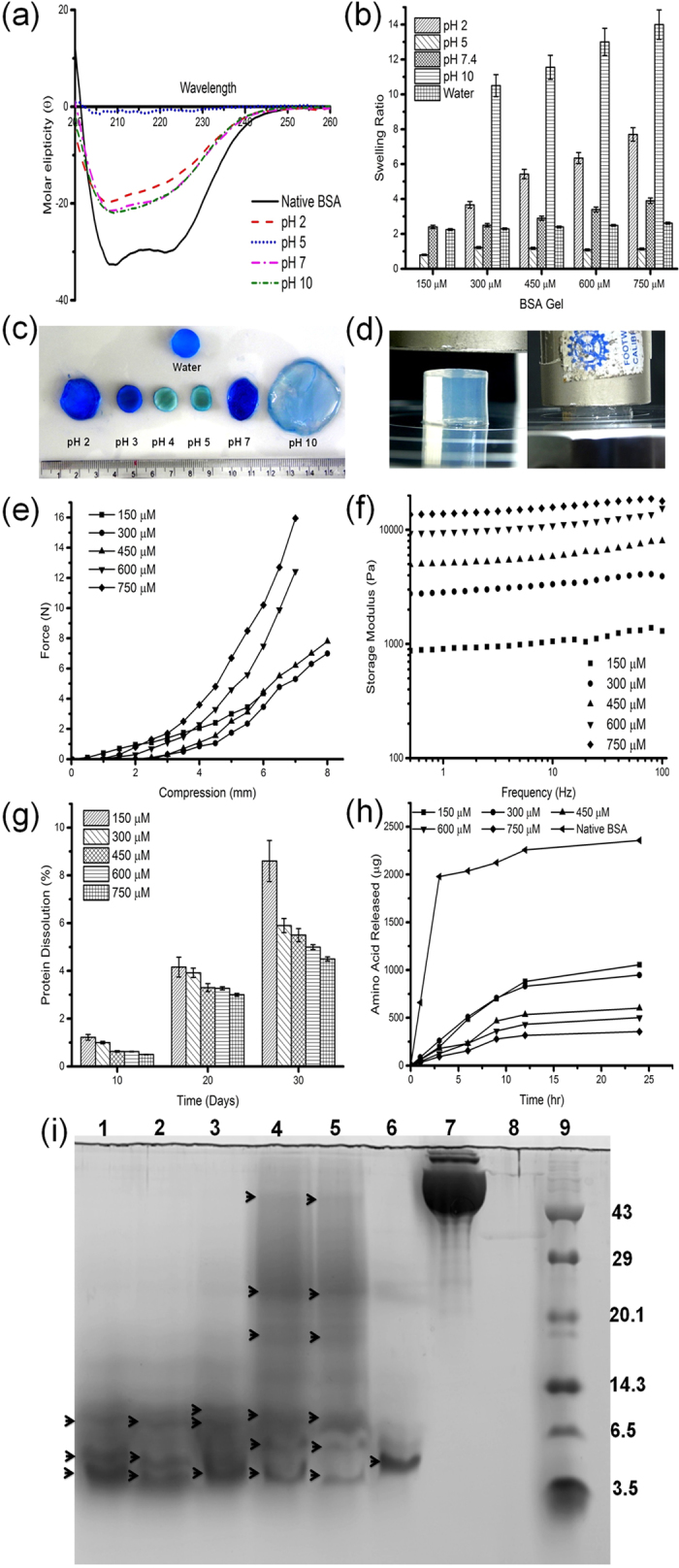
(**a**) Structural conformation of native BSA and oxidatively refolded BSA at different pH. (**b**) The swelling ratio of BSA gel prepared at different concentrations and at different pH environment. (**c**) Pictographic representation of BSA gel swelled at different pH environment and stained with coomassie brilliant blue. (**d**) Pictographic representation of the compression test analysis of BSA gels under room temperature and physiological pH. (**e**) Gel compression studies of BSA gel prepared at different concentrations. (**f**) Rheological profile of BSA gel prepared at different concentrations. (**g**) Stability assessment of BSA gel studied under physiological conditions. (**h**) *In vitro* degradation of BSA gel of different concentration exposed to pepsin and the release of amino acid were quantified using TNBS assay at scheduled time intervals. (**i**) SDS-PAGE profile of pepsin digest of BSA gel prepared at various concentrations. Lane 1 to 5 represents the samples prepared using 150, 300,450, 600 and 750 μM concentrations of BSA gel digest, Lane 6- pepsin digest of native BSA, Lane—7 native BSA and Lane—8 Pepsin alone and Lane—9 molecular weight markers respectively. The black arrowheads represent the different molecular weight pattern of the pepsin digested BSA.

Circular dichroism spectrum was analyzed to ascertain the change in secondary structural conformation of BSA at different pH. The CD spectra of native BSA in PBS exhibits two negative bands in the ultraviolet region at 208 and 222 nm which is a characteristic of α-helix structure of the protein. After assembly, the hydrogel in PBS exhibited reduced band intensity and suggests a change in the secondary structure and reduction in the alpha helical content (Fig. 2a). It can be explained as; during reduction, the protein undergoes disulphide cleavage, which leads to the formation of thiols. In BSA, all the thiols are located in the alpha helical region, during reduction and oxidative refolding the free thiols undergo inter and intramolecular disulphide bridging with other BSA molecules in proxima, which results in the denaturation of the protein structure. Similarly, the effect of pH on the secondary structure of the hydrogel shows similar results for pH 7.0 and pH 10.0. However, at pH 5.0, the hydrogel completely lost its alpha helix (Fig. 2a) and this is a characteristic feature of any protein in its isoelectric point. According to Swanekamp *et al.*^27^, the change in secondary structure and the increase in beta sheet formation responsible for the stability of the hydrogel, which, substantiates the gel strength results observed in the present study.

Swelling study was carried out for all the concentrations of the BSA gel as a function of pH (Fig. 2b). It has been observed that irrespective of BSA concentration, the equilibrium in swelling was attained within 12 to 18 h. In the presence of water, a two fold increase in the weight was observed for all the concentrations, inferred that the concentration of BSA does not have any role in the swelling ratio (Fig. 2b). The reason could be attributed to the neutral charge of the solvent and it is well- known that water is a non-ionic liquid, which does not alter the charges in the protein backbone^10^,^28^. However, with reference to pH, the swelling profile varies considerably as shown in Fig. 2b. When the pH of the environment was reduced to 2.0, about 5 to 8 fold increase in weight was observed with respect to BSA concentration. Similar observations were also made at pH 7.0 and 10.0 (Fig. 2c). The reason may be attributed to the charge distribution of the amino acids. Protonation at acidic pH and deprotonation at alkali pH, impart the positive and negative charges respectively to the amino acids results in high repulsive forces between the BSA molecules and enhances the swelling to the maximum.

The mechanical strength of the hydrogel was evaluated by gel compression analysis (Fig. 2d). All the experiments were carried out at physiological condition at 37 °C. Figure 2e shows the peak force required to break the BSA gel undergoing deformation/compression. BSA gel prepared with 150 μM concentration shows a compressive force of 4.3 N and undergoes only minimum compression of 4.2 mm, reasoned to the improper crosslinking. However, when the concentration of BSA increases, the corresponding increase in crosslinking density (as evidenced from rheology analysis) and a dense network formation offered appreciable strength. BSA gel prepared using 300 and 450 μM concentration shows a maximum deformation (80%). In general, hydrogels behaves as rubber like elastic material, when applying stress, the polypeptide chain undergoes deformation and later undergoes a rapid rearrangement. Similarly, pore size also determines the mechanical strength of the hydrogel, when the pore size decreases (Table 1), the compression force required to break the gel increases. Usually, uniform crosslinking in protein results in even distribution and well connected pores, which act as a barrier and do not allow the crack to propagate easily. In the present study, the maximum compressive force observed suggested the existence of even distribution and well connected pores. However, at higher concentration, though there was an increase in compressive strength, the deformation decreases to 67% due to (i) dense crosslinked structures; (ii) very small pore size; and (iii) reduction in the average molecular weight between the crosslinked network does not allow the gel to escape its water molecules and took maximum force to break the network structure.

The rheological analysis was performed at physiological pH (7.4), 37 °C with 1% strain (since 1% strain falls within the linear viscoelastic region) and by varying the oscillatory frequency. The storage/elastic (G’) and loss/viscous modulus (G”) were calculated. Rheological analysis of native BSA solution at 750 μM solution concentration shows that the loss modulus was greater than the storage modulus (results not shown). The rheological analysis of BSA gel displayed higher storage modulus than the loss modulus and suggested the elastic nature of the hydrogel (Fig. 2f). When increasing the concentration of BSA from 150 to 750 μM, a significant increase in storage modulus was observed. The tan δ value for the BSA gel obtained from 150, 300 and 450 μM albumin concentration decreases from 0.451, 0.363 and 0.278 (Table 1) respectively suggested that the elastic nature of the material increases with an increase in crosslinking density. However, BSA gel prepared at 600 and 750 μM concentration shows an increase in the tan δ value and infers a decrease in the elastic nature of the material with increase in the brittle nature as evidenced in the mechanical compression analysis.

The mesh size and the average molecular weight between the crosslink was calculated based on the peak storage and loss modulus of the BSA gel using affine network model^29^. When the concentration of the BSA increases from 150 to 750 μM (1 to 5%), the mesh size decreased from 14.3 to 6.3 nm, which implies, when the crosslinking density and the protein concentration increases, the porosity decreases that correspondingly decreases the average molecular weight between the crosslinked network as shown in Table 1.

The stability profile of the BSA gel was assessed under two different environments. Figure 2g display the release of proteins from BSA gel when it is exposed to PBS for the period of 30 days. On day 30, BSA gel of 150 μM concentration shows nearly 9% of the protein release and this release might be due to the surface erosion of the BSA gel. By extending the incubation period, the hydrogel imbibes more water and loosens its network, which results in the complete dissolution of the hydrogel (150 μM) in the medium. However, BSA gel of 750 μM concentration displayed higher stability and only less than 6% of protein was released after 25 days of incubation. The results infers the stability of the hydrogel was directly proportional to the concentration of BSA and the stable network between the molecule prevent the dissolution of BSA gel in the medium. Similar kind of observations were made under proteolytic environment (Fig. 2h). After 24 h, BSA gel of 150 μM concentration displayed 48% degradation, however, hydrogel of 750 μM concentration shows only 15% degradation and suggested that increased levels of crosslinking prevents the enzyme substrate interaction and reduces the degradation percentage. Further, the electrophoretogram of the enzymatic digestion of the BSA gel (Fig. 2i) suggests when the concentration of BSA increases from 150 to 750 μM, there was a significant change in the molecular pattern of proteolytic fragments of the BSA gel compared to the native BSA. Native BSA molecule shows a distinguished low molecular weight peptide in the range of 3.5 to 6 kDa. BSA gel prepared in the concentration of 600 and 750 μM shows number of high molecular weight peptides compared to the BSA gel of low concentration. This clearly indicates that during oxidative refolding the protein undergoes inter and intra molecular disulphide crosslinking, which restricts the site for enzymatic cleavage.

In general, porosity, chemical composition and the mechanical properties are the deciding parameters in the preparation of hydrogels for tissue engineering applications. Pore size controls the nutrient diffusion, cellular growth, distribution and vascularisation. Chemical composition facilitates the cell adherence and proliferation. The mechanical property depends on the porosity of the material and it may closely mimic the storage modulus of the native extracellular matrix, which undergoes dynamic stress. All the above said properties can be controlled by the concentration of the constituents and the crosslinking density^30^.

In the present study it has been evidenced that (i) an increase in albumin concentration (150 to 750 μM) increases the polymeric network available for crosslinking, which results in the dense crosslinked network structures with a reduction in the porosity; (ii) increase in albumin concentration increases the hydrogel stability, rheological property, compressive strength and proteolytic resistance. However, the gel compression analysis suggested that the increase in the albumin concentration from 150 to 750 μM increases the gel strength, but decreases the gel deformation when the concentration was above 450 μM. It has been understood that at higher albumin concentration (600 and 750 μM), the hydrogel at a given dimension tend to holds less water molecules and does not allow the imbibed water molecules to migrate from the regions under high load towards the unloaded region and results in high gel strength and low deformation. For delivery of drugs/cells *via* hydrogels, optimum gel strength and deformation is required to resist the *in vivo* stress. The observations made in the present study suggested that hydrogel obtained from 450 μM albumin concentration was found to be optimum for the application studies.

Rat subcutaneous implant model has been employed inorder to assess the *in vivo* degradation and the host tissue response of the hydrogel. Two concentrations were chosen (300 and 600 μM BSA gel) based on the porosity^31^,^32^, and the corresponding tissue response and the biodegradation of the implant materials were observed on day 5, 10, 15, 20 and 30. Throughout the course of the study all the implanted animals were found to be healthy.

**Figure 3:**
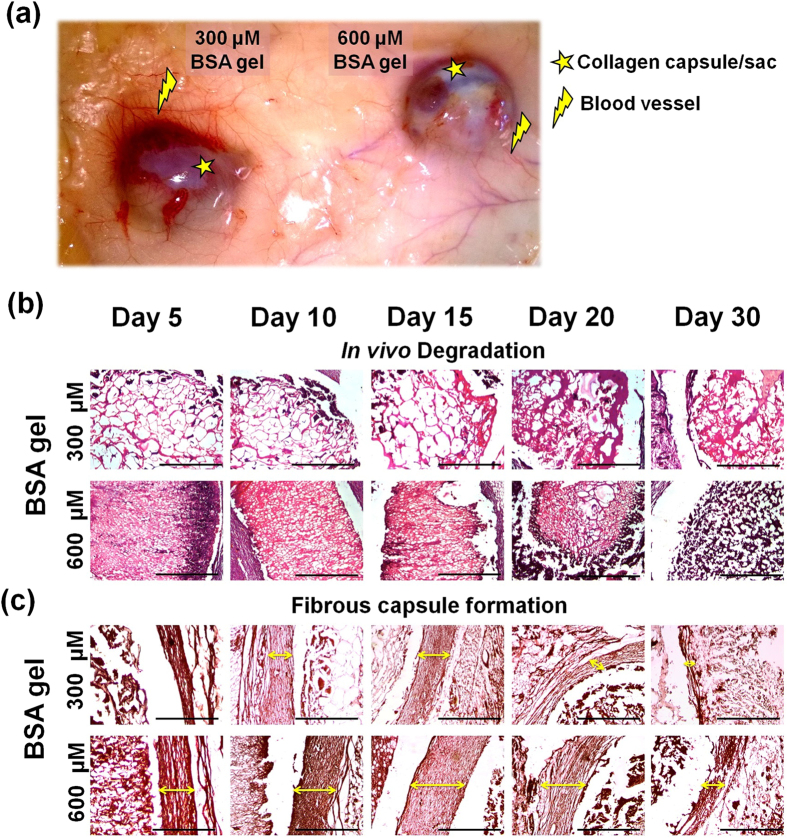
*In vivo* degradation and tissue response analysis of BSA gel indented as a subcutaneous implant in albino rats. (**a**) Explants of BSA gel obtained on day 20, showing lesser collagen capsule and increased blood vessels for 300 μM BSA gel compared to 600 μM BSA gel (**b**) *In vivo* biodegradation assessment of BSA gel prepared at 300 μM (bigger pore size) and 600 μM (smaller pore size) concentration. (**c**) Observations on the fibrous capsule formation around the BSA gel.

Results implied that the implants ensured nil influence on erythema or edema (Supplementary file Table S2) formation in the skin of the animals till the completion of the experimental period. The skin prick test also demonstrates that the subcutaneous implant does not show any loss in the sensitivity of the skin. The explanted hydrogels were intact with the skin and the subcutaneous tissue. Figure 3a, demonstrates the BSA gel (300 and 600 μM) explants obtained on day 20 depicts the vascular growth and collagen fibrous capsule/sac formation. Further, the macroscopic and microscopic observations of the BSA gel showed a slow degradation and complete dissolution within 15 to 20 days and 30 to 40 days for 300 μM and 600 μM BSA gel respectively. (Fig. 3b). Figure 3a,c represents the formation of a fibrous capsule/sac upon implantation. It has been observed that the 300 μM BSA gel provokes less fibrous capsule formation, when compared to that of 600 μM BSA gel. However, as the experimental day proceeds, the reduction in the size of fibrous capsule suggested the degradation and biocompatibility of BSA gel. Discussion on the H & E staining of the the samples (skin-implant-fat layers) has been given in the Supplementary file (Figure S1 and Table S1). According to Rattner^31^ Sussman *et al.*^32^, the porosity of the material determines the collagen capsule formation and vascularity when subjected to the tissue response studies. These authors observed that low porous materials facilitates collagen capsule formation with less vascularization, whereas the reverse was observed with high porous materials. Similar observations were made in the present study on tissue response with both high porous and low porous hydrogels obtained from 300 and 600 μM concentration respectively.

**Figure 4:**
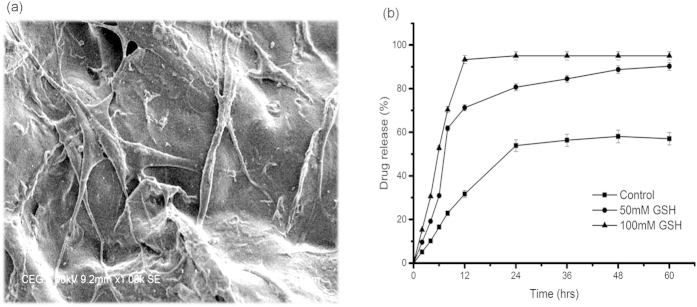
(**a**) SEM micrograph of the BSA gel (450 μM) shows fibroblast cell adherence with elongated spindle shaped cell processes. (**b**) *In vitro* drug release behavior of BSA gel.

Cytotoxicity studies were assessed using NIH 3T3 cells and the experiments were conducted for the albumin hydrogel of 450 μM as a representative sample of the study. The results shows more than 95% of cell viability and this demonstrates that oxidative crosslinking of the BSA does not have any negative impact on cell viability. No significant difference in the cell proliferation was observed when compared to the control (Figure S1) and this implies the non-cytotoxic^30^ nature of the gel. Figure 4a illustrates the scanning electron microscopic image of fibroblast cells adhered to the BSA gel.

Though serum albumin is a blood protein, upon reduction and oxidative refolding it undergoes transformation to a hydrogel and thus necessitates the tests of compatibility. Experiments on hemocompatibility under both indirect and direct contact methods using red blood cells revealed, only 2.5 ± 0.5% hemolysis was observed by the indirect method and 4.5 ± 0.5% with the direct method implying the non-hemolytic nature of the hydrogel.

In order to assess the drug release properties of BSA gel, Tetracycline, a broad spectrum antibiotic was chosen as a model drug. Figure 4b depicts the drug release profile of the BSA gel (450 μM) in the presence of reduced glutathione at different concentrations. It has been observed that under non reducing condition, there was a controlled release of drug and after 24 h more than 50% drug was released. However, under reduced environment, i.e. in the presence of glutathione (50 mM), the drug release was fast and about 60% of the drug was released within 8 h. The drug release percentage increases with the increase in glutathione concentration (100mM) and about 95% of the drug was released within 12 h. The burst release could be reasoned to the reduction of disulphide bonds in the presence of glutathione. These observations suggested that BSA gel as a redox sensitive drug carrier, which is in demand in the case of treatment of cancer cells. According to Ganta *et al.*^33^, Pack *et al.*^34^, Memg *et al.*^35^, reduction sensitive bonds used in bioconjugates add functional properties to the biomaterials particularly for intracellular gene and drug delivery. The degradation and the release of drugs purely based on the reduction of disulfide bonds by GSH^36^. In cancer therapy, the role of disulfide stabilized materials has been studied extensively and thus supports the plausibility of using the redox chemistry involved in the prepration of BSA gel for the treatment of cancer cells.

## Methods

### Albumin hydrogel preparation

#### Step 1: - Reduction of albumin

BSA was reduced according to the following procedure. In brief, BSA (150, 300, 450, 600 and 750 μM) solution was prepared in 0.1 M Tris buffer (pH 9.0) containing 8 M urea and 25 mM glutathione reduced. The reaction was allowed to stir for 1 h at 37 °C.

#### Step 2: - Oxidative refolding of albumin

The reduced samples were then transferred to the dialysis chamber and dialyzed against 0.1 M phosphate buffer pH 6.5 with repeated changes of buffer, under 37 °C open atmosphere. To confirm the complete removal of glutathione, Elman’s assay was performed for the dialyzing medium. Followed by dialysis, the gel material in the dialysis bag was removed and stored at 4 °C.

#### Effect of pH on gel formation

To study the effect of pH on the gel formation, BSA was reduced as described above and reoxidized by dialyzing against different pH environment (pH 3.0, 4.0, 5.0, 6.0, 7.0, 8.0 and pH 9.0) using respective buffers.

#### Scanning Electron Microscopy

The surface morphology of the BSA gel prepared at various concentrations (150, 300, 450, 600 and 750 μM) were assessed for its structural morphology using scanning electron microscope (HITACHI-S3400N) operated at 5 kV. The hydrogels prepared at different concentration were frozen at −80 °C overnight and freeze dried. The cross sections of the freeze-dried samples were placed on the carbon ribbon and gold sputtered. The cross sectional view was observed and captured at different magnification.

### Circular Dichroism

In order to ascertain the change in the secondary structure, the BSA gel obtained at different pH conditions were subjected to Circular Dichroism analysis. All Circular Dichroism (CD) measurements were performed using Jasco J715 spectro polarimeter at room temperature, using a circular quartz cell with a path-length of 0.1 cm. The instrument was calibrated with ammonium d-camphor-10-sulfonate as described by the instrument manufacturer. All CD spectra were measured between 200 and 300 nm with a scanning speed of 100 nm/min. The bandwidth, response time and data pitch was set to 1 nm, 1 sec and 0.5 nm, respectively. All the CD spectra represent the average of three individual scans and all the spectra were solvent subtracted.

### Swelling ratio

The swelling property of the BSA gel was studied by conventional gravimetric procedure. BSA gel was dried at 50 °C for 24 h. After complete drying the pre weighed gel was immersed in double distilled water at 37 °C. Periodically, the swollen gels were taken and the excess surface water was removed using filter paper and weighed until attaining the equilibrium stage. Similarly, the influence of pH on the swelling was estimated by immersing the dried gel in PBS solution prepared at pH 2.0, 5.0, 7.4 and 10.0 respectively. The swelling ratio was calculated from the following equation, swelling ratio = (weight of swollen gel at given time)/weight of dry gel.

### Compression analysis

Mechanical stability, another major feature in demand for the materials was determined for the BSA gel prepared by the reduction- reoxidation process by uniaxial compression tests^37^. In brief, cylindrical hydrogel was prepared with the diameter of 1.5 cm and 1cm height was taken and mounted on the platform of universal testing machine (Instron). The compression strength and the compressive modulus were calculated with the crosshead speed of 10 mm/min and the test was performed at room temperature. All experiments were carried out in triplicates.

### Rheological analysis

Rheological analysis of the BSA gel was carried out using an oscillatory rheometer (Anton Paar Rheometer MCR-301) of parallel plate geometry of 8 mm diameter with 0.1 mm gap. The experiments were carried out at 37 °C using 1% strain and the frequency was varied from 0.1 to 100 rad/s. The change in visco-elasticity was recorded as storage (G’) and loss modulus (G”). The results were plotted as an average of three independent experiments for all the concentrations studied.

According to the rubber elastic theory, the average mesh size (ξ) and the average molecular weight between the crosslinks were calculated using the following formula^29^,

Average mesh size, ξ = (G’N_A_/RT) ^−1/3^

Average molecular weight between the crosslinks, M_c_ = (cρRT/G’)

where G’ is the storage modulus, N_A_ is Avogadro constant, R is the molar gas constant, ρ is the density of water and T is the temperature (K).

### Stability assessment of BSA gel (degradation studies)

Stability, an important requisite property of a hydrogel has been determined for BSA gel prepared in the present study^37^. The study has been performed under two different environments. In the case of first environment, the stability assessment was made in the presence of Phosphate buffered saline and the second environment consists of proteolytic enzyme (pepsin). At scheduled time intervals (3, 6, 9, 12 and 24 h) the degraded samples were subjected to TNBS assay^38^ and SDS-PAGE analysis^39^.

### *In vivo* degradation and foreign body response

*In vivo* degradation and the foreign body response of the subcutaneously implanted BSA hydrogel obtained by reduction- reoxidation process was studied in accordance with the approval guidelines given by Institutional ethical committee, vide no. 466/01a/CPCSEA–IAEC No. 08/02/2011b. Twenty one female albino (Wistar strain) rats with an average weight of 225 ± 5 g were segregated and housed in standard animal cages and fed with pelletized feed and surplus water.

During experiments, animals were anesthetized with ketamine:xylazine (60:10 mg/kg body weight) intraperitoneally. The surgical area was shaved and sterilized with 70% ethanol. For all the experimental animals a paravertebral incision (2.0 cm long and 0.3–0.4 cm depth) was made on the skin using standard surgical blades. With blunt end scissors approximately 2.0 cm wide two subcutaneous pockets were created on the adjacent side. BSA gel prepared using two different concentrations: 300 and 600 μM were chosen as a representative samples for the study and the size of the material was fixed as 10 mm diameter and 50 mm height. The incised wound was closed using absorbable sutures and necessary care was taken for the complete recovery of animals to its healthy condition. The samples were collected on day 5, 10, 15, 20 and 30 of the experimental period. Physical observations on erythema, edema and skin sensitivity were made. On the day of sampling, the test animals were euthanized by cervical dislocation. The implants were collected along with the underlying tissue and stored at 10% buffered formalin for 1 week for histological studies. The tissue sections were processed for hematoxylin and eosin staining (H&E) to access the histomorphological analysis. Picro Sirius red staining was employed to visualize the collagen capsules. Images were captured using Nikon Eclipse 80i microscope and the collagen deposition in the wounded area was calculated using Nikon NIS element D-3.2 software. The sections were further analyzed for blood material interaction, provisional matrix formation, acute/chronic inflammation, granulation tissue and fibrous capsule formation.

### Hemo-compatibility assessment

Hemo-compatibility of the material was studied by both direct and indirect contact assays^39^,^40^ using a representative sample. In the indirect method, hydrogel (450 μM) samples (10 mm dia and 10 mm height) were sterilized by immersing in 70% alcohol and rinsed with sterile PBS and then incubated for the period of 5 to10 days in PBS. At scheduled time intervals, the PBS extract was recovered and treated with 1ml of RBC cells for one hour at room temperature. Followed by incubation, the solution was centrifuged at 3000 rpm for 5 min. The hemolytic potential of the material was recorded by measuring the optical density (OD) of the supernatant at 540 nm. The percentage hemolysis was calculated from the following equation: (OD of positive control–OD of sample)/OD of positive control ×100. According to the percentage of hemolysis the scores were given as hemolytic (>5%), slightly hemolytic (2–5%) and non-hemolytic (<2%). Type I water and PBS were taken as a positive and negative control respectively.

In the direct contact method, 1 ml of blood was exposed to the hydrogel for 1 h at room temperature. Followed by incubation, the samples were centrifuged at 3000 rpm for 5 min. Later, the hemolytic potential was calculated as described in the previous paragraph.

### Cyto-compatibility assessment

Cytocompatibility of the hydrogel was studied using an indirect contact method with NIH-3T3 fibroblast cells^5^,^39^. In brief, BSA gel (450 μM as a representative sample) was incubated with 10 ml of sterile RPMI medium without FBS (fetal bovine serum) for the scheduled period of 5 to10 days. Followed by incubation, the samples were centrifuged and the supernatant with 10% FBS was used for culturing the fibroblast cell. Cell culture plates (96 well) were seeded with 1 × 10^4^ cells per well with the prepared medium and incubated for 48 h at 37 °C with 5% CO_2_. After incubation, the supernatant of each well was replaced with MTT diluted in serum-free medium and the plates incubated at 37 °C for 4 h. After removing the MTT solution, dimethyl sulphoxide was added to each well and aspirated to dissolve all of the crystals and then left at room temperature for a few minutes to ensure the solubility of the crystals. Finally, absorbance was measured at 570 nm using UV plate reader (Epoch, BIOTEK). The results were compared with cells treated with RPMI medium without the hydrogel extract.

To study the morphology of the cell adhered to the BSA gel (450 μM), a cylindrical hydrogel was prepared with the diameter of 1.0 cm and 0.3 cm height and washed with sterile PBS. Later, the hydrogel was placed in the tissue culture plates and the cells were seeded at the density of 4 × 10^4^ cells per well. After 24 h, the cells were fixed using 10% formalin and dehydrated with series grade of alcohol and the cell seeded hydrogel was subjected to SEM observations.

### Drug encapsulation and release profile studies with BSA gel

Drug release profile of BSA gel was studied using Tetracycline as a model drug. Based on the λ_max_ value of tetracycline, a standard graph was plotted with various concentrations. BSA gel of concentration 450 μM (as a representative sample) was prepared and loaded with the drug Tetracycline. Later the hydrogel was immersed in 0.1 M citrate buffer (pH 2.0) and incubated at 37 °C under shaking condition at 100 rpm. The controlled release of the drug was evaluated by measuring the absorbance of the release medium at 360 nm at various time intervals. In addition, experiments were conducted in the presence of glutathione at different concentration in the release medium. In brief, tetracycline loaded BSA gel was kept in the different release medium containing glutathione at various concentrations (0, 50 and 100 mM) and incubated at 37 °C under shaking condition at 100 rpm. After scheduled time intervals, the samples were withdrawn and quantified the released drug using the standard graph.

## Conclusions

Tissue engineering and drug delivery system invite biocompatible materials with responsiveness to the environment. The present study explores the significant observations made during the reduction and oxidative refolding of serum albumin and its transformation into an autogenic material or self-derived material with dual responsive characteristics. It has been observed that concentration of albumin and pH conditions determines the physical, mechanical and biological properties of the hydrogel. The optimum albumin concentration was identified as 450 μM. The redox responsive property of the albumin gel obtained in the present study was found to be suitable for the intracellular drug delivery system, where, the reduced environment is prevalent. The physical, mechanical and biological characterization studies demonstrated, the potency of the albumin hydrogel prepared in the present study meets, the required materialistics property of tissue engineering and drug delivery. The chemistry involved in the preparation of albumin hydrogel invites several design strategies for the preparation of scaffolds, microspheres and nanoparticles.

## Additional Information

**How to cite this article**: Thirupathi Kumara Raja, S. *et al.* pH and redox sensitive albumin hydrogel: A self-derived biomaterial. *Sci. Rep.* **5**, 15977; doi: 10.1038/srep15977 (2015).

## Supplementary Material

Supplementary Information

## Figures and Tables

**Table 1:** The average mesh size, average molecular weight between the crosslinks and viscoelastic property of the BSA gel was calculated based on the maximum peak storage modulus of the rheological data.

CBG gel (wt/v) (%)	G’ (Pa)	G” (Pa)	tanδ G”/G’	ξ (nm)	M_c_ (kg/mol)
1	1385	626	0.451	14.3	17.835
2	4095	1490	0.363	10.01	12.06
3	7880	2190	0.278	8.05	9.404
4	13650	3860	0.282	6.70	7.093
5	18750	8610	0.4592	6.03	6.4
