# Supplementary material for: pH and redox sensitive albumin hydrogel: A self-derived biomaterial
Source: Sci Rep. 2015 Nov 3;5:15977. doi: 10.1038/srep15977 (PMC4630586; doi:10.1038/srep15977)
Supplement: Supplementary Information [file srep15977-s1.pdf]

Supplementary File

pH and redox sensitive albumin hydrogel : A self-derived biomaterial

Thirupathi Kumara Raja. S,[1] Thiruselvi. T,[1]
Asit Baran Mandal,[1] Gnanamani. A, [1]*
[1]CSIR-CLRI
Adyar, Chennai
Tamil Nadu, India

* Author for correspondence

Dr. A. Gnanamani
Microbiology Division
CSIR-CLRI
Adyar, Chennai
Tamil Nadu, India
Email: gnanamani3@gmail.com
Fax: No. 91-44-24912150

Cell culturing and maintenance of RBC and fibroblast cells

For hemocompatiblity studies, RBC cells were used. In brief, human blood (5 ml) was collected and mixed with one ml of anticoagulating medium (Acid citrate dextrose solution). Later, the RBC cells were isolated by centrifuging the blood at 3000 rpm for 5min and the pellet was resuspended in PBS and stored at 4 °C.

For cytocompatability studies NIH 3T3 embryonic mouse fibroblast cells procured from NCCS, Pune, India, used for the present study. The cultures were maintained in DMEM supplemented with 10 % Fetal Bovine Serum (FBS), 200 mM Glutamine, 2 mg/ml Sodium bicarbonate and 1X antibiotic and antimycotic solution. Periodically the medium was replaced. The cells were cultured in tissue culture flasks and incubated at 37 °C in a humidified atmosphere of 5% CO_2. Trypsin at 0.05% was used to detach the cells.

Experimental procedure Stability assessment of BSA hydrogel

Stability, an important requisite property of a hydrogel has been determined for BSA hydrogel prepared in the present study. The study has been performed under two different environmental conditions: (i) in the presence of PBS alone (Mandal et al., 2009) and (ii) in the presence of proteolytic enzymes as summarized by Shalaby & Park (1990).

With respect to the stability assessment in the presence of phosphate buffered saline, hydrogel with 10 mm diameter and 5 mm height was prepared and immersed in 10 ml of phosphate buffered saline (PBS, pH 7.4). The samples were incubated for 30 days at room temperature under shaking (100 rpm) condition with periodical check for the protein release in the PBS. Quantification of the protein release, determined using Bradford assay (at 595 nm) and calculated from the standard graph prepared with native BSA. To prevent any microbial contamination during the prolonged incubation, 0.02 % sodium azide was added to PBS (Mandal et al., 2009).

For the stability assessment in the presence of proteolytic enzymes, 10 mg of oven dried sample of the hydrogel was immersed in 0.1 N HCl solutions and exposed to pepsin (250 U) and the sample was incubated at 37 °C under shaking condition (100 rpm). At scheduled time intervals (3, 6, 9, 12 and 24 hours), the resulting solution was subjected to TNBS assay (Fields, 1972). The assay procedure in brief was summarized in Chapter 3.

In order to analyze the molecular profile of the enzyme digested sample, SDS-PAGE analysis was performed (Laemmli, 1970). In brief, electrophoresis was carried out using 12 % polyacrylamide gel for resolving and 4 % for stacking gel. The length of the separating and stacking gel were of 6 and 2 cm respectively, with a gel thickness of 1 mm. Initially, electrophoresis was performed at a constant voltage of 60 V for stacking and 120 V for separation. Followed by electrophoresis, the gels were fixed in 50 % methanol, 10 % acetic acid for 1 hour before staining in 0.025 % Coomassie brilliant blue G-250 in 10 % acetic acid for 24 hours. The gel was destained using 10 % acetic acid. The destained gel was photographed to study the molecular pattern.

In vivo tissue response analysis of BSA hydrogel

H&E staining of the sections of the samples (skin-implant-fat layers) illustrates the difference in the porous network structures of the BSA hydrogel gel of 300 and 650 µM concentrations respectively. Relatively, large number of inflammatory cells at the interface of the implant suggested the initiation of active biodegradation process. As time passes, the inflammatory cells adhere on the periphery of the hydrogels and infiltrates deep into the implant. The release of proteolytic enzymes by the inflammatory cells degrades the BSA gel network structures resulting with the reduction in the size of the hydrogel as the observational period extended. On day 15, more than 50 % of the BSA hydrogel (300 µM) was degraded and only 20 to 30 % degradation was observed with 600 µM concentration of BSA hydrogel.

The degradation of polymeric network structures ends completely on day 20 for 300 µM and day 30 for 600 µM BSA hydrogel which suggest that the crosslinking density reduces the pore structure of the BSA hydrogel and prolongs the *in vivo* degradation rate.

With respect to the *in vivo* host tissue response analysis, **Table S1** depicts the various parameters observed on different days for the implanted materials. In general, the biocompatibility of the material depends on the physical, chemical and biological nature of the material and the site of implantation (Anderson, 2001; Ratner, 2004). On day 5 of implantation, a clear provisional matrix formation on the periphery of both the BSA hydrogels was observed. The provisional matrix formation provides the basement structure and further initiates several biochemical factors, which mediates the cellular responses for inflammation, neovascularization and infiltration of inflammatory cells. Despite the recruitment of more numbers of inflammatory cells in the surrounding tissues, there was no hemorrhage or necrosis observed.

The remodeling stage begins with the reduction in the blood vessel formation and occasional macrophages (**Figure S2**). Interestingly, no chronic inflammations was observed till the completion of the experimental period. On day 24, a complete dissolution of the BSA gel implant was observed and the basement membrane matrix was restored without any alterations and suggested the high biocompatible nature of the BSA hydrogel. The high interaction of BSA gel with host tissue as evidenced implied the suitability of BSA gel for tissue engineering application as well as tissue regeneration.

Figure S1

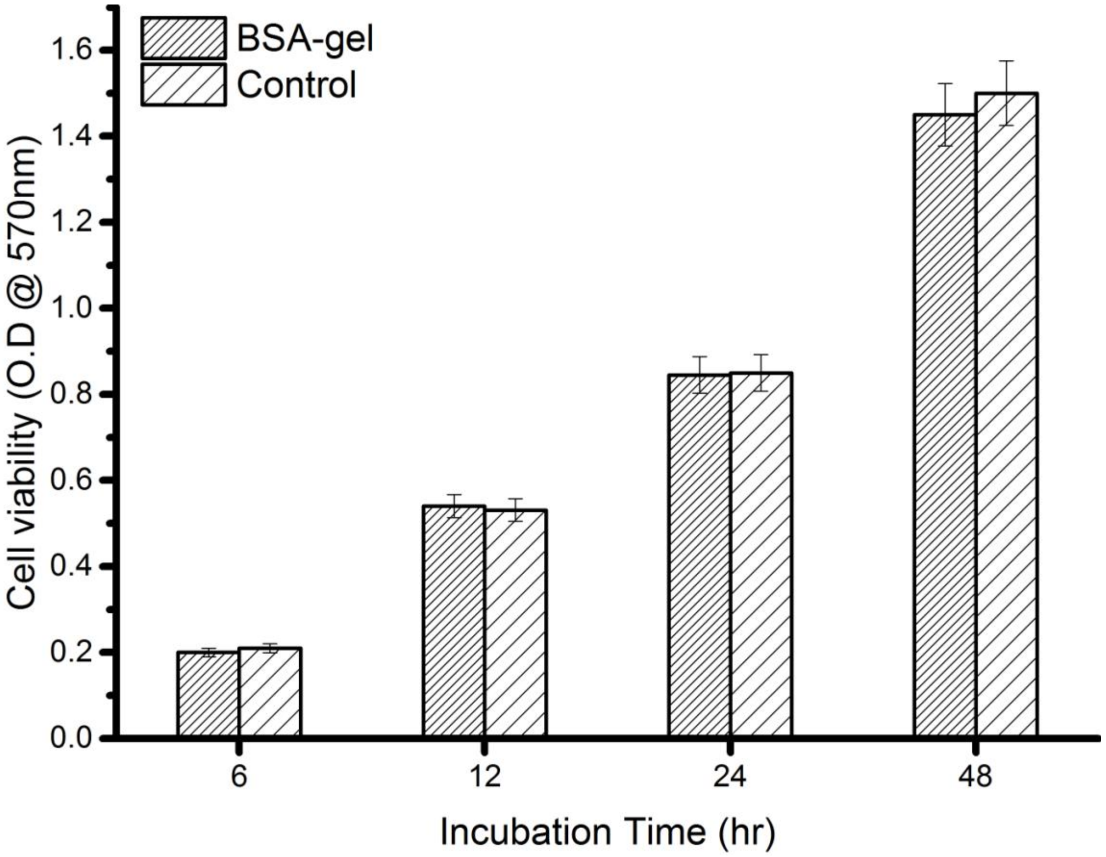

Cytotoxicity studies on BSA hydrogel (450µM) studied at different time periods and quantified using MTT and compared to the control (Media alone).

Figure S2

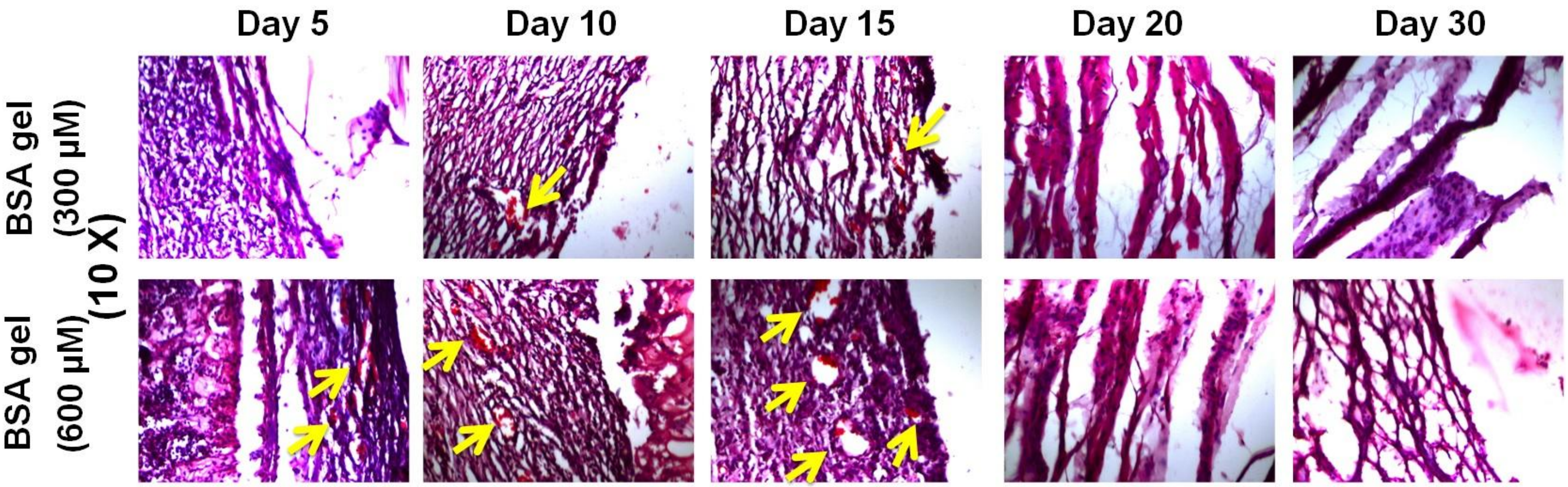

Histopathological observations of BSA hydrogel implant and the tissue response analysis studied using H&E staining. Yellow arrow heads represents the blood vessel formation.

Table S1

In vivo host tissue response analyses of BSA gel on different experimental period.

	**300 µM BSA gel**					**600 µM BSA gel**				
	**Day 5**	**Day 10**	**Day 15**	**Day 20**	**Day 30**	**Day 5**	**Day 10**	**Day 15**	**Day 20**	**Day 30**
**Erythema**	N	N	N	N	N	N	N	N	N	N
**Edema**	N	N	N	N	N	N	N	N	N	N
**Skin sensation**	H	H	H	H	H	H	H	H	H	H
**Macrophages**	N	L	L	N	N	L	M	H	H	M
**Lymphocytes**	L	L	L	L	L	M	H	H	H	M
**Necrosis**	N	N	N	N	N	N	N	N	N	N
**Vascularization**	N	L	L	N	N	N	H	H	M	M
**Fibroblast**	L	L	M	L	N	N	L	M	M	L
**Fibrous capsule**	L	M	M	L	L	M	H	H	M	L

*****Scores are given based on the physical observation and histopathological analysis.

(H-high, M-Medium, L-Low and N-Nil/Non occurrence
